# Supplementary material for: Medullary Thyroid Carcinoma Without Calcitonin: A Case Linking Ultimobranchial Bodies to Tumor Evolution
Source: Pathophysiology. 2025 Oct 23;32(4):56. doi: 10.3390/pathophysiology32040056 (PMC12641662; doi:10.3390/pathophysiology32040056)
Supplement: Supplementary file 1 [file pathophysiology-32-00056-s001.zip › pathophysiology-3649854-supplementary.pdf]

Supplementary material

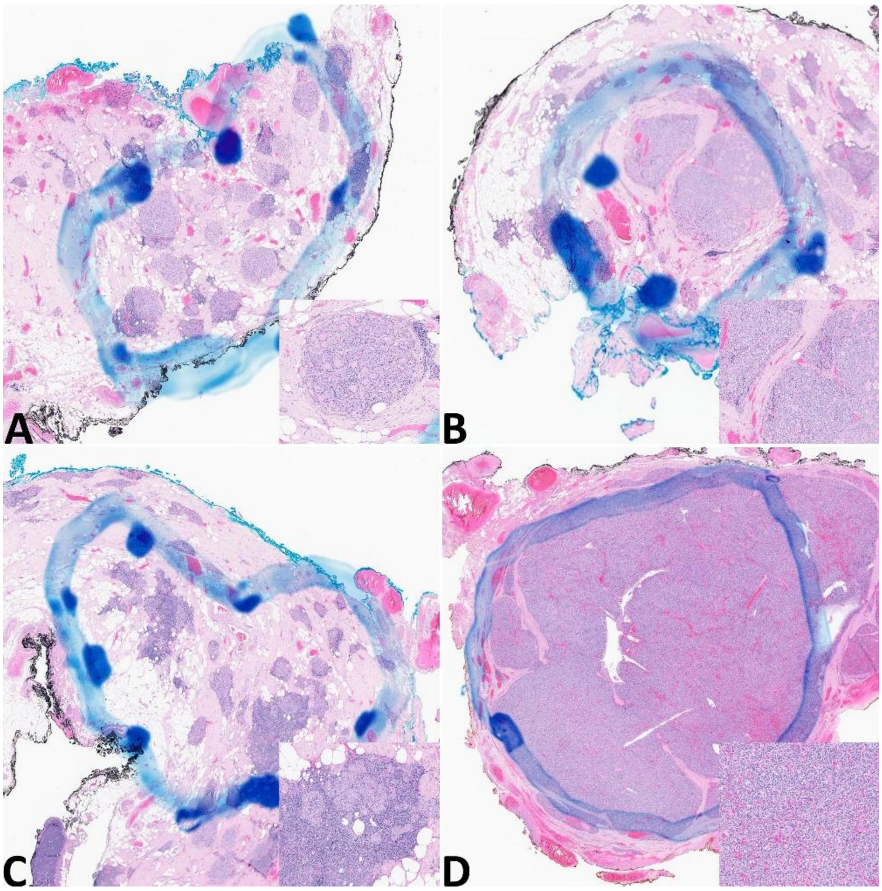

Figure S1

Microdissected targets used for ThyroSeq testing

Tissue targets used for microdissection: A: C-cell hyperplasia, B: C-cell hyperplasia/medullary microcarcinoma, C: Ultimobranchial body remnants within lymphoepithelial islands, D: Medullary thyroid carcinoma

Table S1. Molecular findings for discrete areas of thyroid corresponding to UBB, CCH, CCH/MMC, and MTC. UBB—ultimobranchial body remnants, CCH—C-cell hyperplasia, CCH/MMC—C-cell hyperplasia/medullary microcarcinoma, MTC—medullary thyroid carcinoma.

	Description	DNA (ng/µL)	RNA (ng/µL)	Result
1	CCH	2.62	8.09	MTC POS (C-cell markers 93%)
2	CCH/MMC	2.88	6.35	MTC POS (C-cell markers 98%)
3	UBB	3.80	11.35	MTC POS (C-cell markers 34%)
4	MTC	49.73	84.07	MTC POS (C-cell markers 69%)
